# Spinal Epidural Hematoma after Thoracolumbar Posterior Fusion Surgery without Decompression for Thoracic Vertebral Fracture

**DOI:** 10.1155/2016/6295817

**Published:** 2016-02-18

**Authors:** Tsuyoki Minato, Masayuki Miyagi, Wataru Saito, Shintaro Shoji, Toshiyuki Nakazawa, Gen Inoue, Takayuki Imura, Hiroaki Minehara, Terumasa Matsuura, Tadashi Kawamura, Takanori Namba, Naonobu Takahira, Masashi Takaso

**Affiliations:** ^1^Department of Orthopaedic Surgery, School of Medicine, Kitasato University, 1-15-1 Kitasato, Minami-ku, Sagamihara, Kanagawa 252-0375, Japan; ^2^Departments of Biomedical Engineering and Rehabilitation, Kitasato University School of Allied Health Sciences, Sagamihara 252-0373, Japan

## Abstract

We present a rare case of spinal epidural hematoma (SEH) after thoracolumbar posterior fusion without decompression surgery for a thoracic vertebral fracture. A 42-year-old man was hospitalized for a thoracic vertebral fracture caused by being sandwiched against his back on broken concrete block. Computed tomography revealed a T12 dislocation fracture of AO type B2, multiple bilateral rib fractures, and a right hemopneumothorax. Four days after the injury, in order to promote early orthostasis and to improve respiratory status, we performed thoracolumbar posterior fusion surgery without decompression; the patient had back pain but no neurological deficits. Three hours after surgery, he complained of acute pain and severe weakness of his bilateral lower extremities; with allodynia below the level of his umbilicus, postoperative SEH was diagnosed. We performed immediate revision surgery. After removal of the hematoma, his symptoms improved gradually, and he was discharged ambulatory one month after revision surgery. Through experience of this case, we should strongly consider the possibility of preexisting SEH before surgery, even in patients with no neurological deficits. We should also consider perioperative coagulopathy in patients with multiple trauma, as in this case.

## 1. Introduction

Spinal epidural hematoma (SEH) is a well-known postoperative complication of spinal surgery. Most cases are clinically asymptomatic. Symptomatic SEH usually presents with spinal nerve deficits, including paralysis and acute, severe pain, and usually requires revision surgery. Most published cases of symptomatic SEH occurred after decompression surgery. Cases of symptomatic SEH after spinal surgery without decompression are rare. We present a case of SEH after thoracolumbar posterior fusion without decompression surgery for a thoracic vertebral fracture.

## 2. Case Report

**Figure 1:**
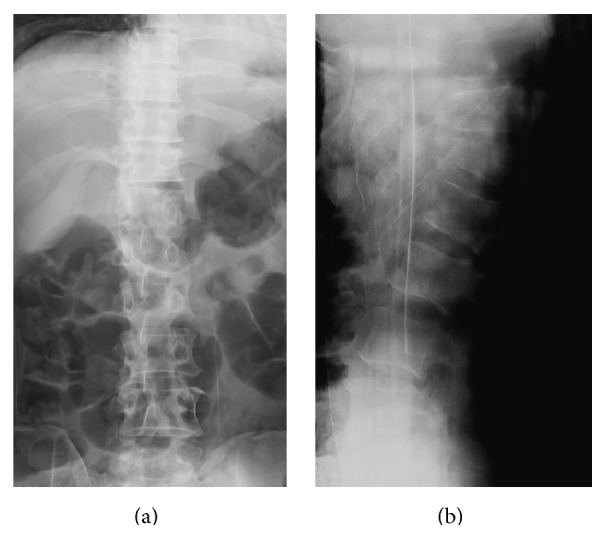
Preoperative radiographs. (a) Anteroposterior (AP) view, (b) lateral view. Fracture and wedge deformity of the 12th thoracic (T12) vertebra.

**Figure 2:**
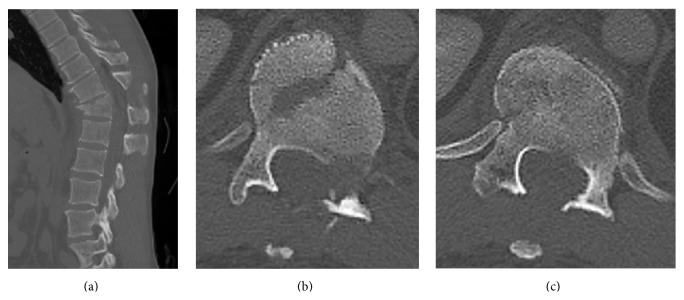
Preoperative computed tomography (CT) images of the spine. (a) Sagittal image, (b) axial image of upper T12, and (c) axial image of lower T12. Anterior, posterior wall, and left pedicle fractures of the T12 vertebra, spinous process fracture of T11, and right T11-12 facet dislocation.

**Figure 3:**
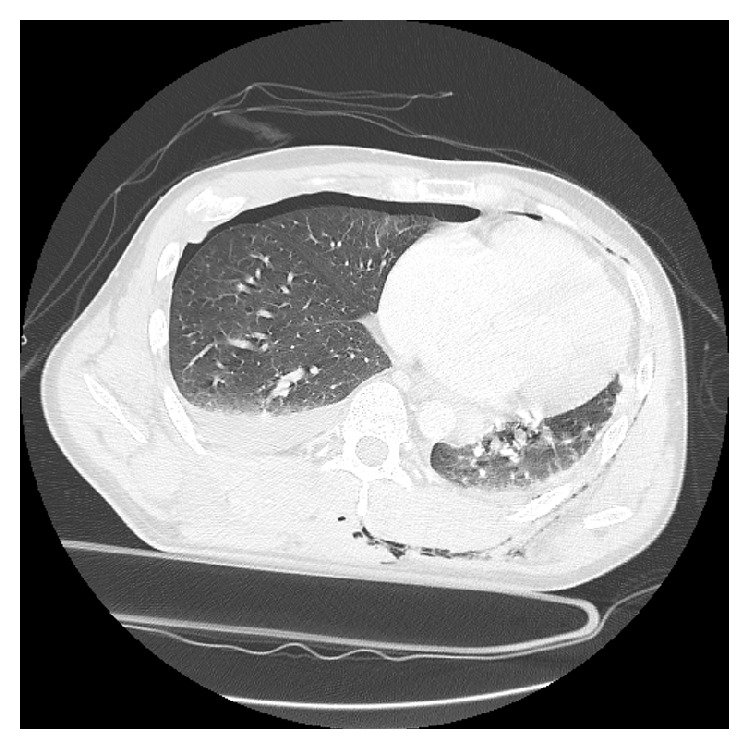
Preoperative CT image of the chest. Multiple rib fractures and a right hemopneumothorax.

A 42-year-old man was injured by being sandwiched against his back on broken concrete block and was hospitalized for severe back pain and difficulty breathing. On initial assessment, the patient was neurologically intact. Radiography revealed a fracture and wedge deformity of the 12th thoracic (T12) vertebra (Figure 1). Computed tomography (CT) revealed anterior, posterior wall, and left pedicle fractures of the T12 vertebra, a spinous process fracture of T11, and a right T11-12 facet dislocation. We diagnosed a T12 dislocation fracture of AO classification type B2 (Figure 2). There were also multiple rib fractures (right ribs 6–8 and left ribs 7-8) and a right hemopneumothorax (Figure 3). We immediately inserted a right chest tube and breathing stabilized. Laboratory results on hospitalization revealed anemia and elevated levels of fibrin degradation products (FDP), D-dimer, and fibrinogen (FIB), but prothrombin time (PT) and activated partial thromboplastin time (aPTT) were within the normal range (Table 1).

To promote early orthostasis and to improve breathing, we planned fusion surgery without decompression for the T12 vertebral dislocation fracture because the patient seemed to be neurologically intact. Four days after the injury, we performed T10-to-L2 instrumented posterolateral spinal fusion with autografted bone. A surgical drain was placed at the time of closure. The operative time was 3 hours and 32 minutes, and the amount of blood loss was 1410 mL because of severe soft tissue damage and the large amount of bleeding from fractures.

**Figure 4:**
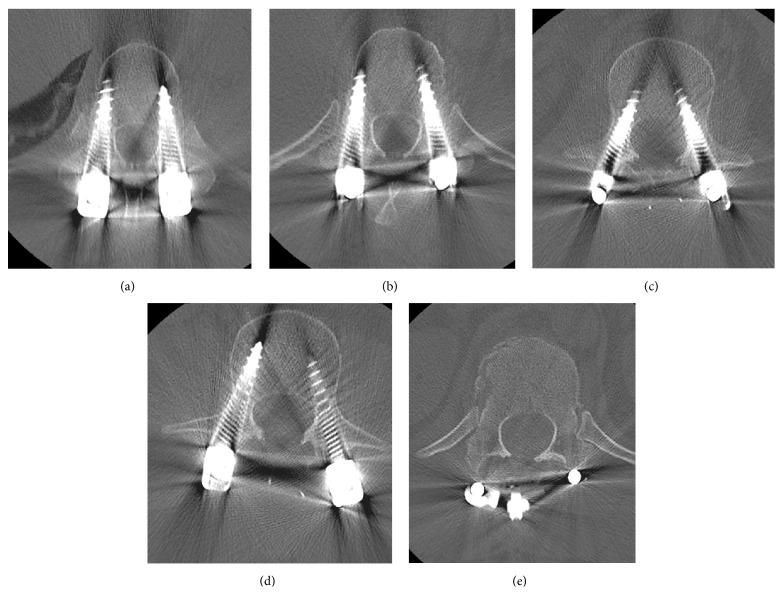
Postoperative CT images of the spine. Axial image (a) T10, (b) T11, (c) L1, (d) L2, and (e) T12. All pedicle screws from T10 to L2 were in correct position, and there were no protruding fracture fragments in the spinal canal at the T12 level from reduction of the dislocated fracture during the operation.

Three hours after the operation, the patient complained of lower leg and back pain. He was found to have motor dysfunction of the lower extremities, with manual muscle testing (MMT) below the iliopsoas muscle graded as 0 to 1. He also had hypalgesia below the T11 level. Immediate CT imaging revealed that all pedicle screws from T10 to L2 were in correct position, and there were no protruding fracture fragments in the spinal canal at the T12 level from reduction of the dislocated fracture during the operation (Figure 4). Magnetic resonance imaging (MRI) was difficult due to instrumentation artifact. Based on these postoperative physical and imaging findings, we diagnosed postoperative SEH. We immediately performed T11 and T12 laminectomy and discovered and removed a large consolidated hematoma (4 × 2 cm) beneath the lamina. Two surgical drains were placed at the time of closure.

**Figure 5:**
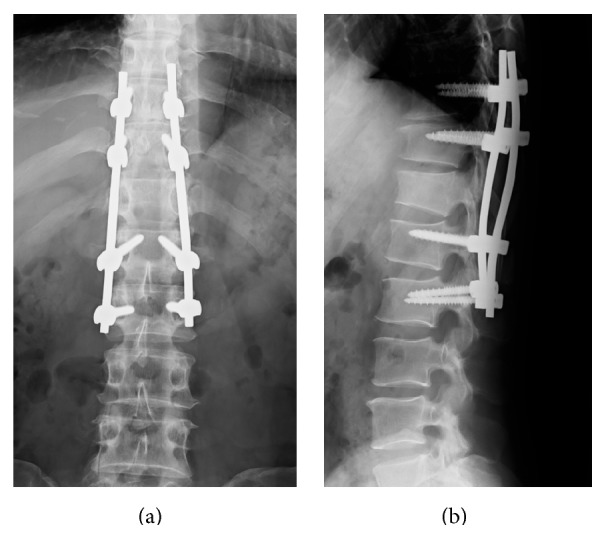
Postoperative radiographs (at final follow-up and 6 months after revision surgery). (a) AP view, (b) lateral view. X-ray at final follow-up revealed a nearly healed T12 fracture.

After the revision surgery, the symptoms gradually improved. He was able to walk with a T-cane on day 20 and was discharged home on day 28. At the final follow-up six months after revision surgery, motor function had fully recovered, but mechanical allodynia persisted and required medication. X-ray at the final follow-up revealed a nearly healed T12 fracture (Figure 5).

## 3. Discussion

SEH usually occurs after spinal surgery, but most cases are clinically asymptomatic. Symptomatic SEH is rare, but postoperative SEH sometimes induces neurological deficits, including sensory, motor, bladder, and bowel disturbances, due to compression of the spinal cord or the spinal nerve root, which may require revision surgery. The complication rate of postoperative SEH requiring revision surgery is reportedly 0.1–3% [1–5]. Most cases of postoperative SEH occur after spinal surgery with decompression. However, there are a few reports of SEH after spinal surgery without decompression. Cain Jr. et al. reported SEH after posterior fusion surgery alone for an L1 burst fracture [6]. However, this was only one case in a series considering postoperative pulmonary embolism, and there was no detailed report. Thus, the current report is the first detailed account of an epidural hematoma that occurred after spinal surgery without decompression.

Several authors reported risk factors for SEH in retrospective and prospective studies. Awad et al. reported that age older than 60 years, the use of preoperative nonsteroidal anti-inflammatory drugs, Rh-positive blood type, more than five operative levels, a hemoglobin <10 g/dL, blood loss >1 L, and postoperative PT-INR (international normalized ratio) >2.0 within the first 48 hours were associated with an increased risk of postoperative SEH [1]. Kou et al. also reported that multilevel procedures and the presence of a preoperative coagulopathy result in a significantly higher risk of developing a postoperative SEH [7]. Sokolowski et al. reported in their prospective study that age greater than 60, multilevel procedures, and preoperative PT-INR were independently associated with postoperative hematoma volume [8]. In summary, perioperative anemia, perioperative coagulopathy, the amount of blood loss, older patients, and multilevel procedures might be among the risk factors for SEH. In this case, preoperative anemia, preoperative coagulopathy, and high volume of blood loss were risk factors for postoperative SEH. In particular, although we did not find abnormal PT-INR postoperatively, we found high levels of other laboratory tests, including FDP, D-dimer, and FIB. Therefore, in evaluating coagulopathy, we should consider not only PT, but also FDP, D-dimer, and FIB.

Regarding the reasons for symptomatic postoperative SEH, the preoperative existence of SEH without neurologic symptoms is possible. SEH after spinal trauma including burst fracture has been reported [9]. Also, SEH or bleeding from vertebra fracture can be trapped in a spinal canal due to the procedure of reduction for dislocation and fracture. However, in this case, we did not consider the preoperative existence of SEH because there were no neurologic symptoms. Based on our experience in this case, in order to diagnose precisely and choose appropriate surgical strategy, surgeons thus should recognize the asymptomatic SEH to be symptomatic after the surgery and may need more detailed radiological evaluation before surgery. In particular, we should evaluate the existence of preoperative SEH by MRI in multiple trauma cases, even in cases without neurologic symptoms. However, detailed radiological evaluation might need more time to be done because of equipmental limitation. Still, the optimal timing of surgery for thoracolumbar fractures and dislocation is still controversy. It has been reported that emergency surgery for thoracolumbar vertebral trauma can improve patients' recovery of neurological function better and also can shorten hospitalization compared with elective surgery [10]. On the other hand, another report indicated that there is no significant neurologic benefit of emergency surgery for traumatic spinal cord injured patients [11]. In addition, it has been reported that the mortality of patients with multiple trauma-associated spinal injury treated by emergency surgery was significantly higher than that in the patients with elective surgery [12]. Based on these reports, in particular, patients who injured thoracolumbar spine with multiple trauma without neurologic deficit might be treated with elective surgery if possible.

Regarding the surgical strategy, whether decompression surgery is necessary in cases of a thoracolumbar dislocation is still controversial. Considering the possibility of bleeding from fractured vertebra or SEH trapped in a spinal canal due to the procedure during surgery, we can add the decompression. However, most cases of postoperative SEH occurred after spinal surgery with decompression. Based on our experience in this case, we need to discuss carefully the necessity to add decompression for thoracolumbar fracture or dislocation with coagulopathy.

## 4. Conclusion

We present a rare case of SEH after thoracolumbar posterior fusion without decompression surgery for a thoracic vertebral fracture. With regard to coagulopathy, we should consider not only PT, but also FDP, D-dimer, and FIB. In addition, surgeons may need to evaluate the existence of preoperative SEH by MRI in multiple trauma cases, even if patients do not have neurologic symptoms.

## Figures and Tables

**Table 1:** Preoperative laboratory data.

White blood cells	12200	/*μ*L
Red blood cells	2.9 × 10^6^	/*μ*L
Hemoglobin	9.6	g/dL
Platelets	14.6 × 10^4^	/*μ*L
Prothrombin time	12.5	sec
Activated partial thromboplastin time	35.3	sec
Fibrin degradation products	21.70	*μ*g/mL
D-dimer	8.96	*μ*g/mL
Fibrinogen	470	mg/dL
C-reactive protein	9.68	mg/dL
